# Formulation and Evaluation of Hybrid Niosomal In Situ Gel for Intravesical Co-Delivery of Curcumin and Gentamicin Sulfate

**DOI:** 10.3390/pharmaceutics14040747

**Published:** 2022-03-30

**Authors:** Viliana Gugleva, Victoria Michailova, Rositsa Mihaylova, Georgi Momekov, Maya Margaritova Zaharieva, Hristo Najdenski, Petar Petrov, Stanislav Rangelov, Aleksander Forys, Barbara Trzebicka, Denitsa Momekova

**Affiliations:** 1 Department of Pharmaceutical Technologies, Faculty of Pharmacy, Medical University of Varna, 84 Tsar Osvoboditel Str., 9000 Varna, Bulgaria; viliana.gugleva@mu-varna.bg; 2 Department of Pharmaceutical Technology and Biopharmaceutics, Faculty of Pharmacy, Medical University of Sofia, 2 Dunav Str., 1000 Sofia, Bulgaria; vmihailova@pharmfac.mu-sofia.bg; 3 Department of Pharmacology, Pharmacotherapy and Toxicology, Faculty of Pharmacy, Medical University of Sofia, 2 Dunav Str., 1000 Sofia, Bulgaria; rositsa.a.mihaylova@gmail.com (R.M.); gmomekov@gmail.com (G.M.); 4 Department of Infectious Microbiology, The Stephan Angeloff Institute of Microbiology, Bulgarian Academy of Sciences, 26 Acad. G. Bonchev Str., 1113 Sofia, Bulgaria; zaharieva26@yahoo.com (M.M.Z.); hnajdenski@gmail.com (H.N.); 5 Institute of Polymers, Bulgarian Academy of Sciences, bl.103 Akad. G. Bonchev Str.,1113 Sofia, Bulgaria; ppetrov@polymer.bas.bg (P.P.); rangelov@polymer.bas.bg (S.R.); 6 Centre of Polymer and Carbon Materials, Polish Academy of Sciences, ul. M. Curie-Skłodowskiej 34, 41-819 Zabrze, Poland, aforys@cmpw-pan.edu.pl (A.F.); btrzebicka@cmpw-pan.edu.pl (B.T.)

**Keywords:** curcumin, gentamicin sulfate, drug delivery, in situ gels, simultaneously loaded niosomes, stimuli-responsiveness, poloxamer, vesicular systems

## Abstract

The current study describes the elaboration of a hybrid drug delivery platform for an intravesical application based on curcumin/gentamicin sulfate simultaneously loaded niosomes incorporated into thermosensitive in situ gels. Series of niosomes were elaborated via the thin film hydration method, evaluating the impact of non-ionic surfactants’, cholesterol’s, and curcumin’s concentration. The formulation composed of equimolar ratio of Span 60, Tween 60, and 30 mol% cholesterol was selected as the optimal composition, due to the high entrapment efficiency values obtained for both drugs, and appropriate physicochemical parameters (morphology, size, PDI, and zeta potential), therefore, was further incorporated into Poloxamers (407/188) and Poloxamers and chitosan based in situ gels. The developed hybrid systems were characterized with sol to gel transition in the physiological range, suitable rheological and gelling characteristics. In addition, the formed gel structure at physiological temperatures determines the retarded dissolution of both drugs (vs. niosomal suspension) and sustained release profile. The conducted microbial studies of selected niosomal in situ gels revealed the occurrence of a synergetic effect of the two compounds when simultaneously loaded. The findings indicate that the elaborated thermosensitive niosomal in situ gels can be considered as a feasible platform for intravesical drug delivery.

## 1. Introduction

The goal to achieve maximum therapeutic efficacy, along with targeted delivery, diminished side effects, and reduced number of administrations, determines the continuous progress in the field of nanotechnology. Great research efforts are focused on cancer therapeutics, where highly beneficial approaches have been reported, such as the PEGylation technique [1], the functionalization of nanocarriers with specific targeting moieties [2], as well as the elaboration of chiral supraparticles [3]. Another interesting and advantageous concept is the development of hybrid drug delivery systems, which, as implied by name, integrate the beneficial characteristics of more than one carrier, leading to enhanced and synergistic therapeutic effects. The present study describes the development of a hybrid drug delivery system for intravesical administration, based on the incorporation of curcumin (Curc) and gentamicin sulfate (GS) simultaneously loaded niosomes into thermosensitive in situ gel. Both compounds were selected as model drugs in our study based on several considerations. From a physicochemical point of view, they exhibit diametrically opposed solubility properties, suggesting their different localization in the niosomal structure, which provides the opportunity to fully exploit the potential of niosomes as a drug delivery platform for both hydrophobic and hydrophilic compounds. In addition, the evaluation of the impact of both drugs on the main physicochemical characteristics of the vesicles, would further expand the conducted research in the field. Regarding their pharmacological activities, both compounds were reported to be successfully applied in the treatment of chronic urinary tract infections [4,5], which serves as a prerequisite to achieve a potential synergistic effect and determines their simultaneous inclusion in niosomes from a therapeutic point of view.

Curcumin (diferuloylmethane) is a natural polyphenolic compound, widely researched in pharmaceutics due its pronounced antibacterial, anti-inflammatory, antioxidant, and antineoplastic activity [6]. Intravesically, the phytochemical is used in the therapy of urothelial carcinomas (non-muscle invasive bladder cancer), and proliferative disorders (cystitis glandularis) [7,8]. According to Xue et al., curcumin can also be successfully applied in cases of chronic urinary tract infections, due to its effect on the expressions of Toll-like receptors’ 2 and 4 mRNA [4]. Still, its propitious therapeutic potential has not been fully exploited in clinical practice due to its disadvantageous physicochemical properties (e.g., low aqueous solubility, pH dependent stability), and biopharmaceutical characteristics (poor absorption, extensive metabolism, and rapid elimination, leading to low oral bioavailability) [9]. In this regard, its inclusion in a nanoscale drug delivery system is a particularly beneficial technological approach, providing the opportunity to overcome the preceding restraints. As previously described, curcumin has been loaded into various types of nanocarriers such as liposomes [10], solid lipid nanoparticles [11], nanostructured lipid carriers [12], and niosomes [13].

Gentamicin sulfate was selected as a second model drug in the current study due to its well-established therapeutic potential in the management of recurrent urinary tract infections (RUTIs) [5], providing the opportunity to explore a potential synergic activity between both compounds. In addition, the aminoglycoside antibiotic is characterized with several drawbacks, such as poor bioavailability, short half-life, and associated side effects (oto/neurotoxicity) [14], which further determines its inclusion in niosomes as an advantageous strategy. In accordance with the above stated, the co-encapsulation of hydrophilic gentamicin sulfate and hydrophobic curcumin conveys a different perspective of our research.

Niosomes are vesicular carriers, which are known to prevent degradation of the incorporated active agents, as well to improve drugs’ stability, solubility, and bioavailability, providing a biocompatible and biodegradable delivery platform with excellent tolerability [15,16]. In addition, they may be further modified with different stimulus-sensitive ligands, as reported by Barani et al. [17], or incorporated into in situ gels, facilitating thereby drugs release in accordance with occurred changes in the microenvironment. Their specific structure—an inner aqueous core and outer hydrophobic bilayer composed of non-ionic surfactants—provides the opportunity to incorporate hydrophobic and hydrophilic drugs individually, as well simultaneously, the latter of which was the first aspect of our study. A series of niosomes (Curc-, GS-, and Curc/GS-loaded) were elaborated, evaluating the effect of different types of non-ionic surfactants, total lipid/drug concentration on the main physicochemical properties—size, size distribution pattern, zeta potential, and entrapment efficiency. The optimal Curc/GS simultaneously loaded niosomes were further incorporated into temperature sensitive in situ gel, in order thereby to exploit the advantages of both systems.

In situ gels are polymeric formulations, which are able to transform from solution into gel in response to changes in the microenvironment’s parameters such as temperature, pH, or ionic concentration [18]. They are characterized with tunable gelling properties by analogy with microgels [19], as well as self-indicating cellulose-based gels [20], which is a highly advantageous characteristic during the elaboration process. In situ gels comprise especially suitable platforms for intravesical application, as the periodic bladder voiding, characteristic for this delivery route, leads to lower drug concentration and short contact time [21]. The latter imposes the necessity of frequent catheter instillations, associated often with patient discomfort, irritation, increased risk of infections, and poor compliance [22]. The transition from sol into gel at body temperature (37 °C), in case of thermosensitive formulations, would contribute to prolonged residence time, whereas the low viscosity of the solution at room temperature facilitates syringeability and injectability. Therefore, a subsequent stage in our study was the evaluation of rheological behavior and the in vitro release profile of optimized hybrid in situ gelling systems. Aiming to achieve a synergistic effect in the treatment of RUTIs (i.e., an improved antibacterial activity) determined the conducted microbiological studies on respective uropathogens, according to ISO 20776 /1-2006 [23].

The aim of the current study was the elaboration of a hybrid delivery platform for intravesical application, consisting of simultaneously Curc/GS-loaded niosomes and a thermosensitive in situ gel. The selected drugs have never been explored as a combination together and are included, respectively, in niosomes, or in situ gels, which presents the novelty of our work. All aspects in the design of the hybrid in situ gel-forming systems are described in detail.

## 2. Materials and Methods

### 2.1. Materials

Curcumin (Curc), gentamicin sulfate (GS), Span 60, Span 80, Tween 60, cholesterol (Chol), Poloxamer 407 (P407), Poloxamer 188 (P188), chitosan (CH) (viscosity 200,000 cps), and o-phthalaldehyde reagent were purchased from Sigma-Aldrich (St. Louis, MO, USA). *Staphylococcus aureus* ATCC 29213, *Escherichia coli* ATCC 35218, *Escherichia coli* (ATCC^®^ 35218TM), both purchased from the American Type Culture Collection (ATCC, Manassas, VA, USA). Ethanol, methanol, chloroform, and all other reagents were of analytical grade.

### 2.2. Methods

#### 2.2.1. Preparation of Curcumin and Gentamicin Sulfate-Loaded Niosomes

Curcumin and gentamicin sulfate-loaded niosomes (individually or simultaneously) were prepared via the thin film hydration method [24]. In brief, non-ionic surfactants and cholesterol in 7:3 to 1:1 molar ratio at total lipid content 30 µmol/mL and curcumin (1.5 µmol/mL) (in case of Curc and Curc/GS niosomes) were dissolved in 10 mL chloroform in a round bottomed flask. The volatile solvent was evaporated at 60 °C and 150 rpm by rotary evaporator (Buchi, Germany), until a thin lipid film was deposited onto the inner surface of the flask. Then, 10 mL purified water or gentamicin sulfate aqueous isotonic solution (40 mg/mL, ≈27 µmol/mL) were added to ensure hydration of the lipid film by rotating the flask under normal pressure at 60 °C and 100 rpm for one hour. A subsequent sonication of niosomal dispersions for 2 min using probe sonicator (Bandelin) was performed; afterwards, the samples were left at room temperature for one hour. The unentrapped drugs were removed by passing the niosomal suspensions through a Sephadex G50 column (Pharmacia, Uppsala, Sweden), equilibrated with saline, and then the purified niosomes were stored at 4 ± 2 °C for successive analysis.

#### 2.2.2. Encapsulation Efficacy

The amount of the entrapped curcumin and gentamicin in niosomes was evaluated spectrophotometrically using a UV–VIS spectrophotometer (Shimadzu UV-1800) after liquid/liquid extraction from the lysed vesicles with chloroform and methanol (1:1 wt:wt) mixture. To the lysed niosomal suspension aliquots of phosphate buffer (pH 6.5) were added and the mixture was vortexed for 10 s and then centrifuged for 5 min at 6000 rpm. The concentration of curcumin in the chloroform layer was evaluated at λ = 427 nm using a calibration curve of Curc in chloroform in the concentration range of 0.001 to 0.01 mg/mL (y = 157.3557x − 0.03842, R2 = 0.996), while gentamicin content was evaluated in the hydrophilic supernatant at λ = 332 nm after derivatization of GS with o-phathalaldehyde reagent (OPA), as described elsewhere [25]. The calibration curve of derivatized GS in methanol was linear in the concentration range 0.002 to 0.075 mg/mL (y = 35 04978x + 0.08687). The measurements were carried out in triplicate and the average values were used.

The entrapment efficiency was calculated as the percentage ratio of the entrapped drug concentration to the total drug concentration and calculated according to the following equation:

(1) $$EE\;\left(\%\right)=\frac{amount\;of\;entrapped\;drug}{total\;amount\;of\;drug}\times 100$$

#### 2.2.3. Size and Size Distribution

The mean size, size distribution, and polydispersity index of the niosomes were determined by dynamic light scattering, using ZetaSizer NanoZS (Malvern Instruments, Malvern, UK), equipped with a 633 nm laser. The values of the hydrodynamic diameter d_h_ were evaluated using back scattering detection at an angle of 175°, and calculated by the Stokes–Einstein equation (Equation (2)):

(2) $$d_{h}=\frac{kT}{3\pi \eta D}◦$$

where *k* is the Boltzmann constant, *T* is the temperature (K), and *η* is the solvent viscosity *η*(H_2_O) = 0.890 × 10^−3^ Pa s at 293 K.

The measurements were performed at 25 °C and the results are presented as ± SD from at least three measurements.

#### 2.2.4. Zeta-Potential

Zeta-potential (ζ-potential) of the prepared niosomal suspensions was determined by electrophoretic light scattering using a ZetaSizer NanoZS (Malvern Instruments, Malvern, UK), equipped with a 633 nm laser at the scattering angle of 175 °C and 25 °C. Zeta-potentials were derived from the electrophoretic mobility using the Smoluchowski equation (Equation (3)):

(3) $$\zeta =\frac{4\pi \eta \nu }{\varepsilon }$$

where *η* is the solvent viscosity, *ν* is the electrophoretic mobility, and *ε* is the dielectric constant of the solvent. The measurements were performed at 25 °C and the results are presented as ± SD from at least three measurements.

#### 2.2.5. Cryogenic Transmission Electron Microscopy (cryo-TEM) Measurements

Cryogenic Transmission Electron Microscopy (cryo-TEM) images were obtained using a Tecnai F20 X TWIN microscope (FEI Company, Hillsboro, OR, USA) equipped with field emission gun, operating at an acceleration voltage of 200 kV. Images were recorded on the Gatan Rio 16 CMOS 4k camera (Gatan Inc., Pleasanton, CA, USA) and processed with Gatan Microscopy Suite (GMS) software (Gatan Inc., Pleasanton, CA, USA). Specimen preparation was performed by vitrification of the aqueous solutions on grids with holey carbon film (Quantifoil R 2/2; Quantifoil Micro Tools GmbH, Großlöbichau, Germany). Prior to use, the grids were activated for 15 s in oxygen plasma using a Femto plasma cleaner (Diener Electronic, Ebhausen, Germany). Cryo-samples were prepared by applying a droplet (3 μL) of the suspension to the grid, blotting with filter paper and immediate freezing in liquid ethane using a fully automated blotting device Vitrobot Mark IV (Thermo Fisher Scientific, Waltham, MA, USA). After preparation, the vitrified specimens were kept under liquid nitrogen until they were inserted into a cryo-TEM-holder Gatan 626 (Gatan Inc., Pleasanton, CA, USA) and analyzed in the TEM at −178 °C.

### 2.3. Preparation and Characterization of Plain and Curc–GS Niosomal In Situ Gels

#### 2.3.1. Preparation of In Situ Gels

Plain and Curc–GS niosomal in situ gels based on thermosensitive polymer Poloxamer 407 and Poloxamer 188 were prepared by the cold method, with or without the inclusion of chitosan [26]. Accurately weighted quantities of Poloxamer 407 (20% *w/w*) and Poloxamer 188 (8% *w/w* and 10% *w/w*) were dispersed under constant stirring in cold (4–6 °C) purified water or niosomal dispersion, and then kept in a refrigerator for 24 h in order to ensure fully dissolution of the polymers. For the preparation of chitosan solution (1% *w/w*), the polymer was dispersed with slight agitation in a 0.5% *v/v* acetic acid solution and left at ambient temperature (25 °C) until the formation of a clear solution. A proper amount of purified water/chitosan solution (1% *w/w*) was further added to Poloxamers solutions to obtain final formulations, maintaining in this way, the polymers’ and drugs’ concentration comparable in all samples.

#### 2.3.2. Evaluation of Gelation Temperature

The gelation temperature of the prepared gels was evaluated by the test tube inversion method as described elsewhere [27]. In brief, 2 mL of tested formulations were placed in a vial (5 mL), which were then immersed in a thermostatically controlled water bath. The temperature was gradually increased by 0.5 °C/min, starting from 20 up to 40 °C, and at each set point the samples were tempered for one minute and then the test tube was inverted at 90°. The temperature at which no flow upon inversion was seen was set as a gelation temperature. For the sake of clarity, the gelation properties of the chosen plain or niosomal in situ gels were further assessed by oscillation temperature sweep experiments as described in Section 2.3.5.

#### 2.3.3. Gelling Time Determination

The test tube inversion method was also used to evaluate the gelling time of the prepared plain or niosomal in situ gels as described by Chaudhari and Desai [28]. Shortly, samples of 2 mL were placed in test tubes (5 mL) and put in a water bath set at 37 ± 0.5 °C. The test tubes were periodically inverted at 90° and the time when no flow was observed, was set as the gelling time. The experiments were performed in triplicate for each sample and the mean value was calculated.

#### 2.3.4. Gel Erosion Time

The time of gel erosion was visually determined as described elsewhere [29]. Briefly, niosomal gel solution (5 mL) was gently transferred into 200 mL of phosphate buffer solution (pH 6.5) tempered at 37 °C in a temperature-controlled water bath without shaking. Thereafter, the time needed for complete dissolution of gel was measured. The measurements were conducted in triplicate.

#### 2.3.5. Rheological Study of Niosomal Thermoresponsive Gels

Dynamic rheological measurements of selected compositions were conducted with a HAAKE MARS 60 rheometer. The tests were performed in CD mode (controlled deformation) with a parallel plate sensor system (top plate diameter = 20 mm; gap = 1 mm). Three runs of each sample were conducted. The gelation process was assessed in oscillation temperature sweep experiments. Storage (G′) and loss (G″) moduli were measured at constant deformation and frequency (ɣ_0_ = 0.003; f = 1.000 Hz) in the 25–45 °C temperature range (20 data points). The sample was equilibrated for 180 s at given temperature before obtaining a data point. Prior to the temperature sweep measurements, amplitude and frequency sweep tests were made to establish the linear viscoelastic range for polymer gels. The formation of gel as a function of time at the physiological temperature (37 °C) was also investigated by using oscillation time sweep tests. In these experiments, aqueous solutions, equilibrated at 25 °C, were abruptly heated in the instrument to 37 °C and then the temperature was kept constant for given time. Storage and loss moduli were determined at ɣ_0_ = 0.003 and f = 1.000 Hz. The oscillation amplitude sweep tests were carried out at 37 °C at a frequency of 1 Hz in the ɣ_0_ range from 0.001 to 10.

### 2.4. In Vitro Drug Release

In vitro release profiles of curcumin and gentamicin sulfate from niosomes and in situ gels prepared thereof were assessed using the dialysis tube method [30]. In the release experiment, 2 mL of the niosomal suspensions or niosome loaded in situ gel were transferred into a dialysis tube closed at one end with a cellophane dialysis membrane (7.065 cm^2^ exposed area, MWCO 12,000–14,000, Sigma-Aldrich, Steinheim, Germany). The dialysis tube was then immersed into an outer vessel containing 90 mL of phosphate-buffered saline (pH 6.5) [22]. А pH value of 6.5 was selected based on the estimated average pH of urine in humans [31]. The dissolution medium was constantly stirred (200 rpm) and maintained at 37 °C ± 0.5 °C throughout the experiment using a circulating water jacket (Huber, Germany). At appropriate time intervals, 1 mL aliquots were withdrawn from the outer aqueous solution and replaced with an equal volume of fresh release medium. Drugs content was evaluated using UV–VIS spectrophotometer (Shimadzu UV-1800) at 427 nm for curcumin and 332 nm for gentamicin sulfate after derivatization with OPA reagent. The experiment was performed in triplicate.

### 2.5. Stability Evaluation

Stability evaluation of the optimal niosomal suspensions and the in situ thermoresponsive gel prepared thereof was performed in terms of size and size distribution and drug content after storage of the samples at 4 °C for one month.

### 2.6. Evaluation of Antibacterial Activity

#### 2.6.1. Bacterial Strains and Culture Conditions

The antibacterial activity of the niosomes was evaluated on the following two bacterial strains: *Staphylococcus aureus* (ATCC^®^ 29213TM) and *Escherichia coli* (ATCC^®^ 35218TM), originating from the American Type Culture Collection (ATCC, Manassas, VA, USA). The strains were cultured under aerobic conditions at 37 °C in Trypticase Soy Agar/Broth (TSA-# M1968 /TSB-# LQ508, Himedia, India). All experiments were performed in Mueller Hinton broth (MHB, #M0405B, Thermo Scientific-Oxoid, Hampshire, UK).

#### 2.6.2. Determination of Minimal Inhibitory Concentrations

The minimal inhibitory concentrations (MIC) of the niosomes were determined by the broth microdilution method (BMD) following ISO 20776/1-2006 [23]. Briefly, 50 µL twofold serial drop dilutions of the niosomes in MHB were prepared in 96-well plates. Gels loaded with curcumin, gentamicin or combinations thereof were tested in parallel. Each concentration was repeated threefold. Gentamicin (#15750060, ThermoFisher Scientific, Waltham, MA, USA) was used as positive control. MHB served as negative control. The bacterial suspension was prepared in MHB from an overnight liquid bacterial culture first at optical density of 1 × 108 CFU/mL (OD600, 0.5 McFarland) and diluted further to a density of 5 × 10^5^ CFU/mL. A volume of 50 µL of the latter was added to each well of the plates excepting the negative control. The plates were incubated at 37 °C for 24 h. Thereafter, the plate wells were examined for bacterial growth and lowest inhibiting drug concentration was determined as MIC. The requirements of EUCAST (European Committee on Antimicrobial Susceptibility Testing) for their MICs were followed for discussing the results (EUCAST 2021) [32]. PBS served as a negative control.

#### 2.6.3. Checkerboard Assay

The checkerboard BMD test was used for the in vitro evaluation of combinations between curcumin and gentamicin loaded in niosomes. The hybrid niosomal in situ gels were mixed in a 96-well plate following the classical schema of the checkerboard assay [33], whereby serial twofold drop dilutions were prepared in a two-dimensional fashion to include all combinations (42/plate in our case) within a specified clinically relevant range for Curc and GS. The result was evaluated by the BMD test as described above.

The combination effects were determined using the FIC (fractional inhibitory concentration) methodology. FICs were calculated by comparing the MIC of each gel loaded either with curcumin or with gentamicin alone to the MIC of that gel in the combination (MICC). The FICs were calculated and interpreted as follows [34]:

(4) $$FIC\;\left(A\right)=\frac{MIC_{C}\;\left(A\right)}{MIC\;\left(A\right)}$$

(5) $$FIC\;\left(B\right)=\frac{MIC_{C}\;\left(B\right)}{MIC\;\left(B\right)}$$

where *FIC* means fractional inhibitory concentration, *A* stands for drug *A* (gentamicin), and *B* is drug *B* (curcumin).

(6) $$\sum FIC=FIC\;\left(A\right)+FIC\left(B\right)\;$$

Synergy was defined as Ʃ*FIC* ≤ 0.5, indifference—as 0.5 < Ʃ*FIC* ≤ 4, and antagonism—as Ʃ*FIC* > 4. Some investigators [34] consider compounds additive when 0.5 < Ʃ*FIC* ≤ 1, which was adopted for this study.

#### 2.6.4. Redox (Dehydrogenase) Activity Assay for Bacterial Cells

The cell redox (dehydrogenase) activity of treated bacteria was measured at the end of the MBD and the checkerboard assays [35]. The MTT dye is reduced in living cells by the membrane-located bacterial enzyme NADH–ubiquinone reductase (H+-translocation) to non-soluble violet crystals of formazan. For this aim, 10 µL MTT dye (stock 5 mg/mL in PBS) was added to each well of the plates containing 100 µL of the respective sample. Plates were kept for 120 min at 37 °C. The resulting formazan crystals were dissolved with an equivalent volume of organic solvent (5% formic acid in 2-propanol). The absorbance was measured at λ = 550/690_ref_ nm (Absorbance Microplate Reader Lx800, BioTek Instruments Inc., Santa Clara, CA, USA) against a blank solution containing the respective volumes of MHB, MTT, and solvent. The inhibition in the metabolic activity of the treated bacteria was calculated as fraction of the untreated control.

### 2.7. Evaluation of Cytotoxicity of Curcumin and Gentamicin-Loaded Niosomes and the In Situ Thermosensitive Gels Thereof

#### 2.7.1. Cell Lines and Culture Conditions

The in vitro cytotoxicity of elaborated empty or Curc/GS-loaded niosomes and in situ gels thereof was evaluated on normal mice fibroblasts (CCL-1^TM^, NCTC clone 929, American Type Culture Collection—ATCC, Manassas, VA, USA), while the antiproliferative activity of the free and nanoformulated curcumin (niosomes and the thereof obtained hybrid in situ gel delivery systems) was assessed against malignant human tumor T-24 cell line (bladder carcinoma origin) obtained from the German Collection of Microorganisms and Cell Cultures (DSMZ GmbH, Braunschweig, Germany). T-24 cells were cultivated according to protocol instructions of the supplier in a growth medium RPMI-1640 supplemented with 10% fetal bovine serum (FBS), 5% L-glutamine, and incubated under standard conditions of 37 °C and 5% humidified CO_2_ atmosphere. CCL-1 cells were maintained following the recommendations of ISO 10993-5, Annex C (ISO 10993-5:2009 2017) [36]. Briefly, they were cultured in Eagle’s Minimal Essential Medium (#MEM-A, Capricorn^®^, Munich, Germany), supplemented with 10% heat inactivated horse serum (#HOS-1A, Capricorn^®^, Munich, Germany) and 2 mM L-glutamine (#G7513, Sigma-Aldrich, Steinheim, Germany) under the same culture conditions as the other cell lines.

#### 2.7.2. MTT Colorimetric Assay

The in vitro cytotoxicity of the newly designed formulations was evaluated using a validated methodology for assessing cell vitality known as the Mosmann MTT method. The test is colorimetric and measures the activity of mitochondrial enzymes reducing the yellow dye MTT (3- (4,5-dimethylthiazol-2-yl) -2,5-diphenyltetrazolium bromide) to violet formazan crystals.

Exponential-phased cells were harvested and seeded (100 µL/well) in 96-well plates at the appropriate density of 1.5 × 10^5^ for the adherent T-24 cells. Following a 24 h incubation, cells were treated with serial dilutions of the Curc/GS-co-loaded niosomes and their hybrid in situ gel formulations in respect to curcumin concentration. Following exposure time of 72 h, filter sterilized MTT substrate solution (5 mg/mL in PBS) was added to each well of the culture plate. A further 1–4 h incubation allowed for the formation of purple insoluble precipitates of the formazan dye. The latter were dissolved in isopropyl alcohol solution containing 5% formic acid prior to absorbance measurement at 550 nm. Collected absorbance values were blanked against MTT and isopropanol solution and normalized to the mean value of untreated control (100% cell viability).

#### 2.7.3. Statistical Methods

The experimental data were processed using non-linear regression analysis in the GraphPad Prism^®^ software program, on the basis of which the semi-logarithmic “dose-response” curves were constructed and the corresponding half-inhibitory concentrations (IC_50_) of the screened compounds were calculated.

## 3. Results and Discussion

The aim of this study is the development of a novel hybrid delivery system, namely, niosomal temperature sensitive in situ gel for intravesical instillation of curcumin and gentamicin sulfate. In this regard, the studies were conducted in two stages: obtaining optimal in composition niоsomes simultaneously loaded with curcumin and gentamicin sulfate and their subsequent formulation into a temperature sensitive in situ gel. To the best of our knowledge, this is the first report on preparation of curcumin and gentamicin sulfate-co-loaded niosomes and the stimulus sensitive in situ gels thereof.

### 3.1. Preparation and Characterization of Curcumin and Gentamicin Simultaneously Loaded Niosomes

A series of niosomal formulations were prepared by the thin film hydration method, evaluating the effect of the formulations’ constituents (e.g., type of surfactants (SF), cholesterol concentration), as well curcumin’s content on the main physicochemical characteristics of the vesicles. The opposite nature of both drugs towards water, the strictly hydrophobic curcumin (logP = 3), and the hydrophilic nature of gentamicin sulfate (logP = −3.1) suggests their different localization in the niosomal compartments. Therefore, as a presumptive part of the niosomal membrane, respectively affecting the vesicles’ integrity, the impact of curcumin’s content was evaluated, while preserving a constant concentration of gentamicin sulfate hydration solution (26.86 µmol/mL) in all samples.

As illustrated in Table 1, the type of surfactant (samples: S1, S2, S3, and S4) influences the main physicochemical properties of the vesicles. The highest entrapment efficiency values for the two drugs were obtained in sample 4 (S4), composed of equimolar mixture of Span 60 and Tween 60. The resultant HLB value of 9.8, corresponds to both the hydrophobic and hydrophilic nature of the selected model drugs, facilitating their inclusion in the niosomal structure. In addition, the larger hydrophilic head group of Tween 60 (compared to Span 60 and 80) may increase the solubilization of curcumin via the possible formation of hydrogen bonds with the phenolic groups of the compound. Similar potential interaction was reported also by Junyaprasert et al., elaborating ellagic acid-loaded niosomes [37]. The hydrophilic nature of Tween 60 contributes also to the observed higher entrapment efficiency values for gentamicin sulfate (S4 and S5) as compared to studied hydrophobic surfactants. Our results are in agreement with a study conducted by Abdelbary and El-gendy, according to which the highest entrapment efficiency for gentamicin sulfate was achieved using Tween 60 as surfactant [38]. However, the successful use of Tween 60 as a membrane forming constituent is predetermined by an appropriate cholesterol concentration, since its highly hydrophilic properties lead to the formation of micelles instead of bilayer structures (S3). The latter necessitates the increase in cholesterol level in order to assemble a vesicular structure (S5). In this regard, it is worth to consider cholesterol’s role also in the arrangement of the membrane bilayer, along with its well-known membrane stabilizing properties. The niosomal formulation S5 exhibited similar entrapment efficiency value for gentamicin sulfate compared to S4, but at the same time an approximately two times lower EE% for curcumin. Therefore, as suitable surfactants for the simultaneous encapsulation of curcumin and gentamicin sulfate, Span 60 and Tween 60 were selected in an equimolar ratio. To derive an optimal composition, the next step in our research was to evaluate the impact of cholesterol’s and curcumin’s concentrations on the main physicochemical characteristics.

As shown in Table 1, the higher cholesterol content (40 mol:mol%, S6) determined the increase in vesicles size compared to S4, while having a disadvantageous effect on the entrapment efficiency of both drugs (more pronounced negative impact on curcumin EE%). The enhanced hydrophobicity of the bilayer membrane could lead to derangement of the vesicular structure; thus, the observed size enlargement may serve as a compensating mechanism to impart stability of the vesicles [39,40]. The estimated lower curcumin entrapment at 40 mol:mol% may be due to the possible competition between cholesterol and the hydrophobic compound, as both are integral constituents of the bilayer membrane. Furthermore, the less rigid structure, associated with lower cholesterol concentration may also contribute to the inclusion of lipophilic drugs [41]. As evident from the data, presented in Table 1, the increase in curcumin concentration had a disadvantageous effect on the entrapment efficiency values for the phenolic compound, even more the twofold increase in curcumin content determined a decrease in the EE% values with approx. 25% (S4 and S9). A possible explanation might be the reaching of the saturation limit, afterwards curcumin precipitates, which may lead to lower entrapment. The entrapment efficiency for GS was not substantially affected by curcumin’s content, which further suggested their different localization in niosomal compartments. However, curcumin’s concentration highly affects vesicles size and zeta potential. At 30 mol% cholesterol content (S4, S8, S9, and S10) the biggest vesicles size was obtained for the empty niosomes and, respectively, the smallest-for the solely curcumin-loaded niosomes. This may be attributed to the inclusion of the hydrophobic compound in the bilayer membrane, which leads to a more compact vesicular structure, as confirmed by the DLS analysis. The vesicles’ size was also influenced by gentamicin sulfate inclusion. The increase in size from 251 to 388 nm (S9 and S8, respectively) indirectly indicates the successful entrapment of hydrophilic GS.

Zeta potential values of elaborated niosomes were ranged from −9.4 to −15.8 mV. The obtained negative values even in case of using non-ionic surfactants were widely discussed in the literature. They may occur as result of the ionization of free groups located onto vesicular exterior, as well due to the orientation of hydroxyl groups towards water and associated alterations of ionic charges [42]. The highest zeta potential values were reported for the blank niosomes and the lowest values for the Curc/GS sulfate simultaneously loaded niosomes based on equimolar ratios Span 60 and Tween 60 and 30 mol% cholesterol. The decrease in zeta potential values may be due to the free drug spread in the aqueous phase or in the diffusion layer [9].

**Figure 1:**
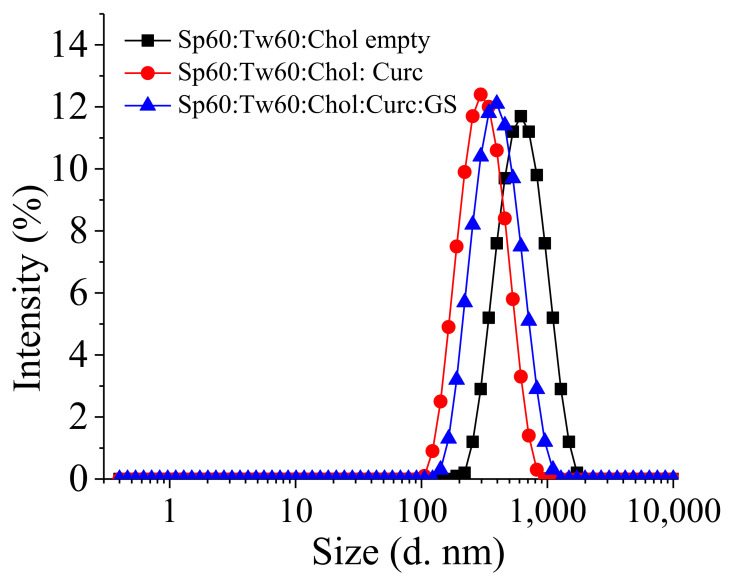
Size distribution of Tw60:Sp60:Chol either empty or simultaneously loaded with Curc and GS. Total lipid ratio is 30 μmol/mL, where Tw60:Sp60:Chol (3.5:3.5:3 mol/mol).

Based on the conducted studies, as an optimal composition for subsequent inclusion into in situ gels was selected, the formulation S8, based on Span 60, Tween 60, and cholesterol (3.5:3.5:3 molar ratio), was characterized with the highest entrapment efficiency values for both drugs and lowest PDI, suggesting narrow size distribution (Figure 1).

### 3.2. Cryo-TEM

**Figure 2:**
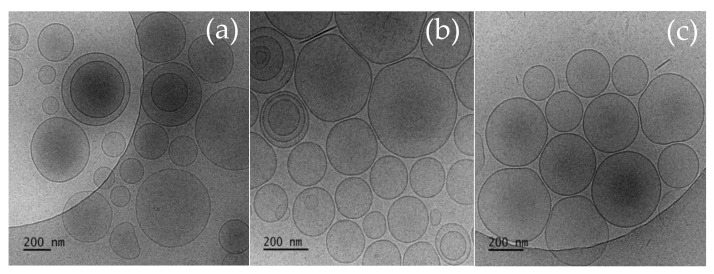
Cryo-TEM images of niosomes composed of Tw60:Sp60:Chlo 3.5:3.5:3 (mol:mol): (**a**) Empty niosomes; (**b**) Curcumin solely loaded vesicles; and (**c**) Curc/GS-co-loaded niosomes. Bars = 200 nm.

The morphology of the empty and Curc and GS sulfate-co-loaded niosomes at optimal surfactants: Chol ratio (7:3) was investigated using cryo-TEM and the representative images are depicted on Figure 2.

As evident from the cryo-TEM images, the chosen preparative method yields the production of well-defined spherical vesicles with intact membranes of 4–5 nm thickness. The empty niosomes (Figure 2a) are characterized with variable size in the range of 80 to 550 nm with average size of approx. 450 nm. The incorporation of curcumin into niosomes is accompanied by a decrease in size and size variability compared to the empty vesicles, so the reported average size of curcumin-loaded vesicles is approximately 250 nm (Figure 2b). The additional encapsulation of gentamicin is associated with an increase in niosomal size (mean size approx. 300 nm) (Figure 2c). These findings correlate well to the size and size distribution data obtained by DLS experiments. Although well-separated intact vesicles dominated in all samples, a small fraction of irregularly shaped aggregates as well as small disc-shaped structures were observed. DLS data also show the presence of a concomitant fraction of particles much smaller than niosomes, but their amount (as intensity, volume, and number of distribution) is less than 1% compared to the main niosomal fraction.

### 3.3. Preparation and Characterization of Niosomal Thermo-Responsive In Situ Gels

#### 3.3.1. Preliminary Evaluation of Gelling Properties of Plain and Hybrid Niosomal In Situ Gels

The second stage of this study was the preparation of hybrid niosomal temperaturesensitive in situ gels based on Curc/GS-co-loaded niosomes (formulation S8) and thermoresponsive polymer Poloxamer 407 (P407). Poloxamer 407 was chosen as the main component of the in situ gels due to its unique reversible thermogelling properties with a T_sol-gel_ transition temperature depending on the polymer concentration [43]. In addition, P 407 possesses an excellent toxicological profile and good mucoadhesive properties. For plausible intravesical application an optimal thermoresponsive gel should be characterized with a gelling temperature in the range 30-35 °C, shorter time for gelation and prolonged gel erosion time [44]. In accordance with the above stated, we evaluated a series of niosomal in situ gel-forming formulations with varying combinations of the polymers: P407, P188, and chitosan. The gels were prepared using the cold method at different polymer’s concentration to find the best formulation with optimal temperature and capacity of gelation suitable for intravesical application (Table 2).

All of the prepared plain in situ gels are clear, colorless viscous solutions at 20 °C, and undergo gelation upon a temperature increase at a certain degree. As evident from the data presented, the formulations composed solely of P407 undergo gelation at room temperature (25 °C or even lower) as the concentration of the polymer increases. The observed tendency could be explained by the mechanism of gel formation of P407. Being an amphiphile tri-block polymer in an aqueous solution at certain concentration (so called critical micellar concentration (CMC)), P407 unimers start to self-associate into spherical micelles and this process is more pronounced as the temperature increases due to the dehydration of poly(propylene oxide) (PPO) blocks and easier micelle formation [45]. At sufficient concentrations, micelles tend to interact with each other and form well-ordered structures—the onset of gel formation [46,47]. At this perspective, higher poloxamer 407 concentration leads to a higher number of micelles, and hence a lower T_sol-gel_ temperature.

To optimize the gelation parameters (time and temperature of gelation), we have prepared in situ gels based on a combination of P407 (at constant concentration of 20%) and varying concentrations of other polymers such as P188 and chitosan. The two polymers were chosen due to their beneficial biological properties, such as biocompatibility, mucoadhesivity, excellent toxicological profile, and low-immunogenicity [48,49].

As evident from the presented results, the addition of P188 leads to an increase in the temperature of gelation. The addition of 8% P188 showed an optimal T_sol–gel_ of 33 °C and very fast gelation (Table 2). This formulation is also characterized with good gel erosion time of approx. 4 h. The increase in the concentration of P188 from 8% to 10% is associated with further increase in the temperature of gelation reaching 40 °C and a higher gelation time. In an attempt to increase the time for gel erosion, chitosan was added in a concentration of 10% or 20% (Formulations G5 and G6). As evident from the data presented, the addition of 10% or 20% chitosan solution leads to a decrease in the temperature of gelation to 31 °C or 30 °C respectively. However, no significant change in the gel erosion time was observed.

**Figure 3:**
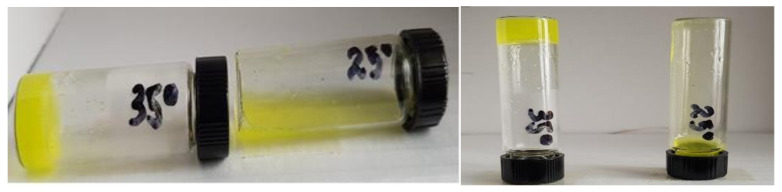
Curc-GS niosomal thermosensitive in situ gel at 35 °C and at ambient temperature.

Based on the results obtained the formulations composed of 20% P407 and 8% P188 with or without addition of a 10% chitosan solution (formulations G3 and G5) were chosen as optimal gel compositions. Using these fixed concentrations of the polymers, hybrid niosomal in situ gels were prepared (formulations G7 and G8). The prepared hybrid niosomal in situ gels appear as yellow-colored translucent viscous solutions at room temperature (Figure 3). The gelling characteristics of niosomal in situ gels are presented in Table 2. As evident from the results, changes in the gelling properties were not observed upon niosomal incorporation leading to the conclusion that the main impact on the gelation behavior of the in situ thermo-responsive gels have the type and concentration of the polymers rather than the presence of niosomal vesicles.

#### 3.3.2. Rheological Studies of Selected Plain and Curc/GS-Loaded Niosomal In Situ Gels

**Figure 4:**
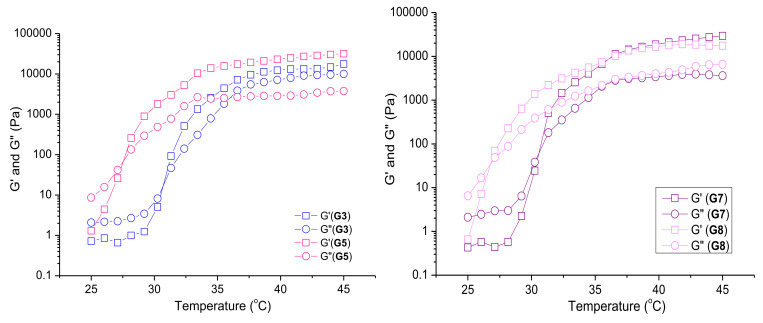
Dependence of storage (G′) and loss (G″) moduli on temperature for pure polymer formulations, G3 and G5, on the left side, and niosome-containing formulations, G7 and G8, on the right side.

Dynamic rheological measurements were used to investigate in more detail the sol-to-gel transition phenomena of selected formulations—G3, G5, G7, and G8. The temperature sweep test was performed at experimental conditions allowing the temperature of material to reach an equilibrium before the next increase in temperature by the rheometer. In this experiment, the temperature was varied in steps of 1 °C with 3 min of equilibration time at each data point. Figure 4 shows the variations in G′ and G″ as a function of temperature. The samples based on P407/ P188 blend (G3 and G7) exhibited the typical for Pluronic systems phase diagram with three well defined regions [50]. The first region corresponds to the liquid state, where the viscous part is dominant (G″ > G′) and both values of the storage and loss moduli are small. At these temperature intervals, polymer micelles, comprising a poly(propylene oxide) core and a poly(ethylene oxide) corona co-exist with the unimers. In the second region, G′ and G″ increase gradually and crossover at given temperature. This is attributed to the increase in the number of micelles with the temperature rise, leading to overlap of micelles and slight entanglement of the chains (clusters of micelles). Thе material behaves as a soft gel. In the last region, G′ is notably higher than G″, the two moduli are several orders of magnitude larger than the corresponding values from the first region, and are also temperature independent. This state is defined as hard gel, characterized by closely packed micelles into a three-dimension network structure. The presence of niosomal carriers in the P407/P188 gel has a minor effect on the sol–gel transition properties. Niosomes, like the micelles, are spherical nanoaggregates, which are randomly incorporated within the micellar clusters without changing the mechanism of gel formation. The crossover point of the two systems was registered at an identical temperature (31 °C), and G′ of the two hard gels at a physiological temperature was about 10 kPa. On the other hand, the addition of high molar mass chitosan (G5, G8) shifted the temperature at which G′ equals G″ to a lower value (27 °C), probably due to pronounced entanglement between the linear chitosan macromolecules and the micellar coronae. Such a bridging effect has been observed for PEO homopolymer having macrochains longer than the hydrophilic segments of F127 micelles [51]. A slight strengthening of the plain gel was found, unlike the niosome-loaded hard gel (G8), which exhibited storage modulus similar to G′ of P407/ P188 gel (G7).

**Figure 5:**
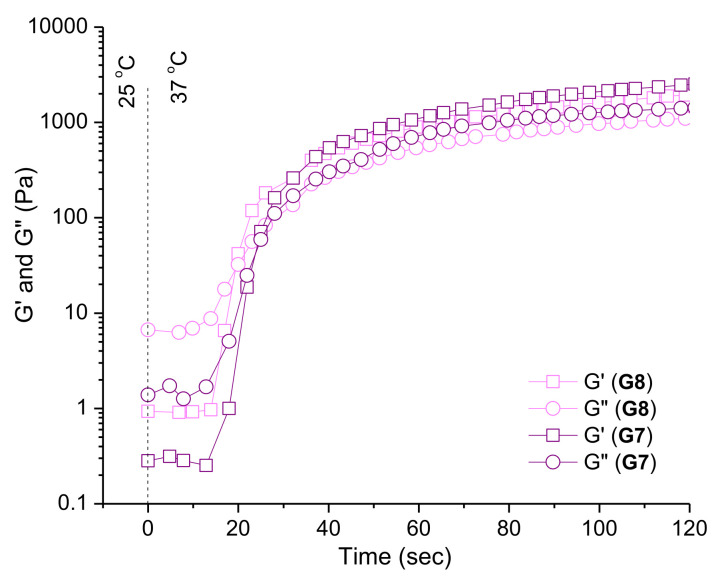
Variation in storage (G′) and loss (G″) moduli as a function of time at abrupt heating of niosome-containing formulations, G7 and G8, from 25 to 37 °C.

Temperature-responsive properties of the elaborated in situ gel formulations were studied also by oscillation time sweep tests. We focused on determining the time required for transition from a liquid state to a hard gel. The test is based on an abrupt heating of the samples (G7 and G8) from room temperature (25 °C) to body temperature (37 °C) in the rheometer. The sol-to-gel transition started within 20 s, while hard gel behavior was found after additional 15 s (Figure 5).

The gelation process, primarily dictated by the properties of PEO-b-PPO-b-PEO block copolymers, begins when a critical temperature of material is reached (T_sol-gel_). At the reported experimental conditions (1 mm gap), approximately 20 s were needed to transform the Newtonian liquid into a soft gel, which then underwent additional structural changes to yield a hard gel for the total time of 35 s. Тhe presence of chitosan accelerated the process by 2 s.

**Figure 6:**
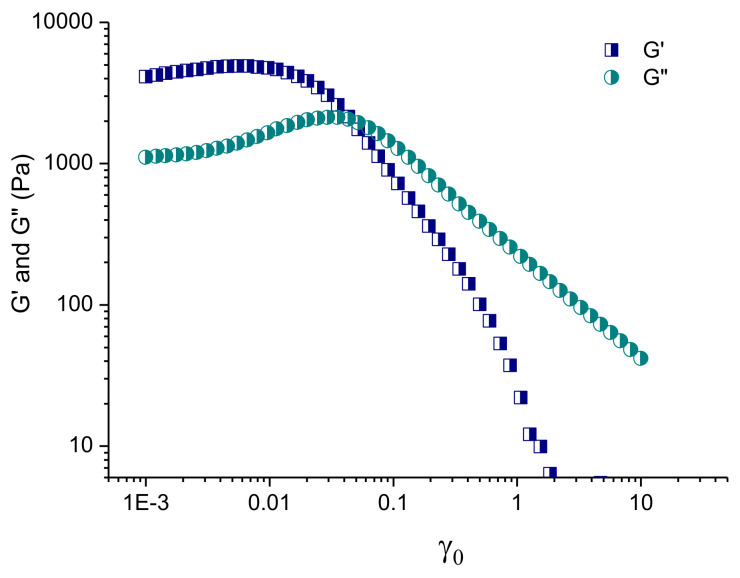
Variation in storage (G′) and loss (G″) moduli as a function of the strain amplitude for niosome-containing in situ gel formulation, G8, at 37 °C.

Figure 6 illustrates the results of the strain sweep test of niosome-containing in situ gels, performed at 37 °C. At a very small strain, both G′ and G″ were nearly constant and G′ was much higher than G′’. As already mentioned, this is a typical hard gel behavior dominated by the elastic component. With the increase in strain, the system firstly underwent a slight shear thickening and, after an overshoot of G″, a shear thinning. The effect of shear thinning is associated with a breakup of the close-packed structures at γ_o_ ~ 0.01, and subsequent sliding of micellar layers in the flow direction [50]. Such behavior is of practical importance, as the change from a highly elastic to a viscous material, when a high shear stress is applied, might affect the rate and mechanism of drug release from in situ gels.

### 3.4. In Vitro Release of Curcumin and Gentamicin Sulfate from Niosomes and In Situ Gels

**Figure 7:**
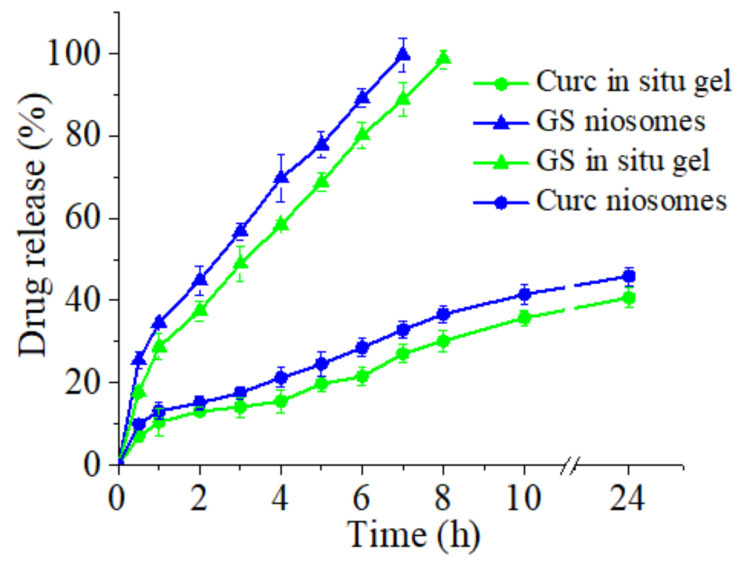
In vitro release profiles of Curc and GS from Curc/GS-co-loaded niosomes (S8) and from hybrid niosomal in situ gel (G7) in PBS (pH 6.5) at 37 °C.

In vitro release profiles of curcumin and gentamicin sulfate from selected niosomal and in situ gel formulations are presented in Figure 7. Curc/GS-loaded niosomes based on Span 60, Tween 60, and cholesterol (3.5:3.5:3 molar ratio) were selected as the optimal composition, due to their favorable physicochemical properties (high entrapment efficiency for both drugs, narrow size distribution), therefore, were further incorporated into in situ gelling system. As shown in Figure 7, the release of the two drugs from niosomes was initially rapid (i.e., burst effect), followed by sustained release until the end of the experiment. The biphasic pattern, illustrating this process, is characteristic for most of the nanoscale drug delivery systems (including niosomes) and is reported also by other authors [52,53]. A significant influence on the in vitro release profile are the physicochemical properties of the co-loaded drugs. The hydrophilic GS exhibits a higher release rate in comparison to hydrophobic curcumin—a tendency maintained also in the in situ gel formulations. Regarding the impact of the drug delivery platform on release process, it should be noted that the incorporation of Curc/GS-loaded niosomes into a gelling system at a physiologically relevant temperature (37 °C ± 0.1 °C) leads to retarded dissolution of both drugs. The decreased release rates are primarily determined by the obstructive effect of the gel structure, which hampers drugs’ diffusion process. A similar trend is reported also by other authors [54]. Additionally, it is worth noting, that the release profiles of co-loaded drugs from both niosomal suspension and gel are identical, which indirectly indicates the well-preserved integrity of the vesicles.

### 3.5. Stability Evaluation

**Figure 8:**
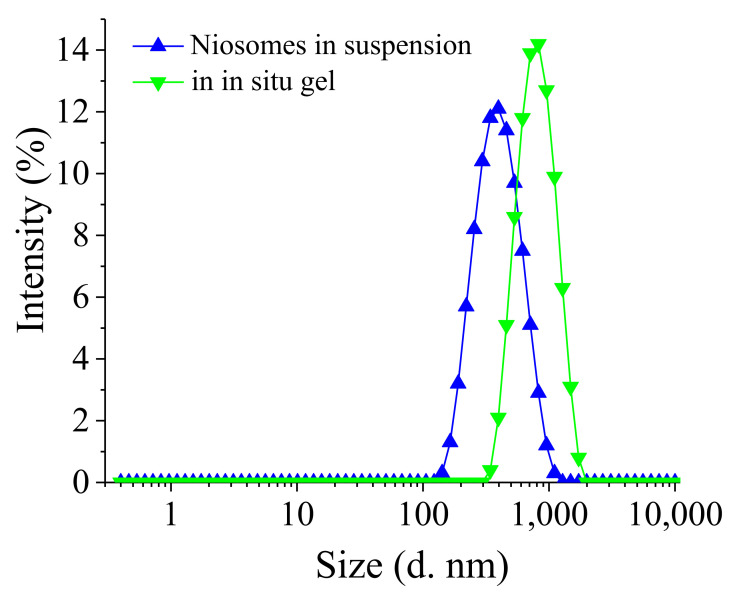
Size distribution of Curc/GS simultaneously loaded Tw60:Sp60:Chol (3.5:3.5:3 mol:mol) niosomes in suspension and after their formulation in in situ gel. DLS measurements of in situ gel were made after sufficient dilution with distilled water.

Physical stability studies were performed to evaluate the effect of storage conditions on the physicochemical properties of the optimal niosomal suspension and in situ gels thereof. The selected formulations were kept for 30 days at 4 ± 2 °C and the results are presented in Table 3. Representative size distribution histograms are depicted in Figure 8. As evident from the data shown, no significant differences in the evaluated physicochemical properties and drug content during the storage period were observed.

The results presented in the table showed that the incorporation of niosomes into in situ gels did not compromise their physical stability. Only a slight increase in the niosomal size (less than 10%) and hardly any change in the PD indices were observed. This indicates that the increase in the niosomal size is probably due to polymer adhesion on the niosomal membrane rather than the aggregation or fusion of the niosomes themselves. Moreover, no significant changes in niosomal size and size distribution patterns were observed upon storage. The hybrid in situ gel formulation also shows good physical stability upon storage as no change in the gelation temperature and drug content was observed. Even more, it can be seen that curcumin and gentamicin sulphate content in the niosomal in situ gel was higher preserved at the end of the experiment, compared to this in the niosomes. These finding confirms the sufficient physical stability of the elaborated drug delivery platforms.

### 3.6. Antibacterial Activity of In Situ Gels

The antibacterial activity of selected hybrid in situ gels (G7 and G8) based on simultaneously loaded Curc/GS niosomes was evaluated vs. free drugs against Gram (+) *S. aureus* and Gram (-) *E. coli* bacteria. As presented in Table 4, the susceptibility of the test strains to gentamicin was consistent with the clinical break points of EUCAST (EUCAST 2021)–0.25 mg/L for *S. aureus* and 2 mg/L for *E. coli*. The minimal inhibitory concentration of free Curc applied in the form of ethanol solution was 50 mg/mL for the reference staphylococcal strain used, which is in line with other reports in the scientific literature [55].

The MICs of Curc and GS loaded into niosomes and incorporated thereafter in in situ thermosensitive P407/P188 gels led expectedly to an increase in their MICs due to the gradual release of both substances from the gels (Figure 7 and Table 4). The in vitro release profiles of the two systems reveal that after 24 h approx. 45% of the loaded amount of curcumin was released (Figure 7). The MIC of curcumin in gel with chitosan was determined to be 40 mg/L for the *S. aureus* strain and to be 80 mg/L for the *E. coli* strain. When a gelling system without chitosan was applied, the curcumin MIC for both test strains was 80 mg/L (Table 4). The release of gentamicin was almost 100% (Figure 7) after 8 h. The MICs of gentamicin-loaded niosomes formulated into gels were 1 and 4 mg/L for the tested *S. aureus* and *E. coli* strains, respectively. The addition of chitosan did not have an influence on the activity of GS. The higher MICs of GS in the gelling system could be explained with the gradual release of the drug from the gel. The concentration-dependent bacterial killing effect of GS [56] is a plausible reason to load higher concentration of gentamicin into the gels and to expect higher MIC values as compared to the free drug.

In order to evaluate the combination effects between Curc- and GS-loaded niosomes after their formulation into in situ gels, and to see if such combinations will lead to diminishment of the antibacterial GS and Curc concentrations, a checkerboard assay was performed. For this aim four additional thermosensitive P407/P188 gels with or without chitosan based on single loaded curcumin or gentamicin sulfate niosome were prepared. Analysis of the results obtained from checkerboard assay revealed that curcumin applied in the concentration range of 0.3–20 mg/L (depending on the test strain and the presence or absence of chitosan) diminished twofold the MIC of gentamicin (from 1 to 0.5 mg/L for the staphylococcal strain and from 4 to 2 mg/L for the *E. coli* strain), resulting in ∑FIC = 0.5, which can be determined as synergistic effect (Table 4). The metabolic activity of the treated bacteria after exposure to the synergistic combinations was very low and varied from 0.55% to 10% depending on the strain and gel system composition. However, the detection of the remaining dehydrogenase activity in the samples indicates the bacteriostatic effect of the synergistic combinations. The MICs of GS niosomes included in gels remained below or at the clinical breakpoint, which indicates the clinical relevance of the tested hybrid niosomal in situ gels.

**Figure 9:**
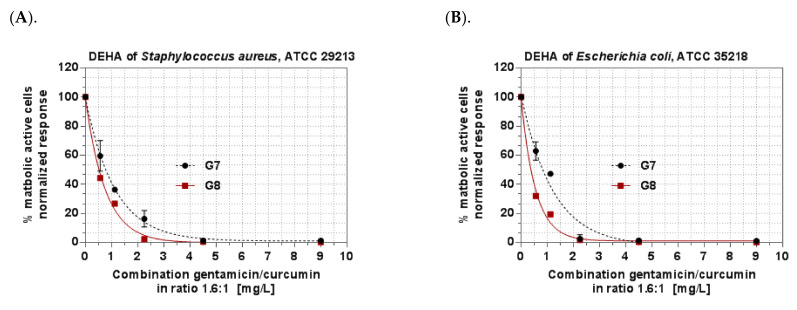
Metabolic activity of *S. aureus* and *E. coli* treated with a combination of curcumin and gentamicin in the ratio 1:1.6 wt:wt. (**A**). DEHA activity of *Staphylococcus aureus* after treatment with combination gentamicin/curcumin in ratio 1.6:1. (**B**). DEHA of *Escherichia coli* after treatment of combination gentamicin/curcumin in ratio 1.6:1. Legend: DEHA—dehydrogenase activity. The concentrations plotted on the X-axis represent the sum of the concentrations of gentamicin and curcumin.

Based on the results from the checkerboard assay, the optimal for antibacterial effect Curc to GS 1:1.6 (wt:wt) ratio was found and the hybrid niosomal in situ gels with or without chitosan were prepared based on the simultaneously Curc/GS- (1:1.6 wt:wt) loaded niosomes. The results obtained for the MICs and MBCs of gels based on the Curc/GS-co-loaded niosomes confirmed the efficacy of the synergistic combinations predicted in the checkerboard assay. The graphs on Figure 9 represent the concentration-effect relationship of the in situ Curc/GS niosomal gelling systems on the metabolic activity of both tested bacterial strains. The full inhibition of the bacterial dehydrogenase activity corresponds to the MBCs of the gels. The staphylococcal strain was found to be more sensitive to the combination than the *E. coli* strain regarding the MIC values. The MBC values were found to be equal to both strains with the exception of the MBC value of gel containing chitosan (G8) on the tested *E. coli* strain, which is twofold lower. Based on the results obtained, the addition of chitosan increases antibacterial activity against the *E. coli* strain.

### 3.7. Cytotoxicity

**Figure 10:**
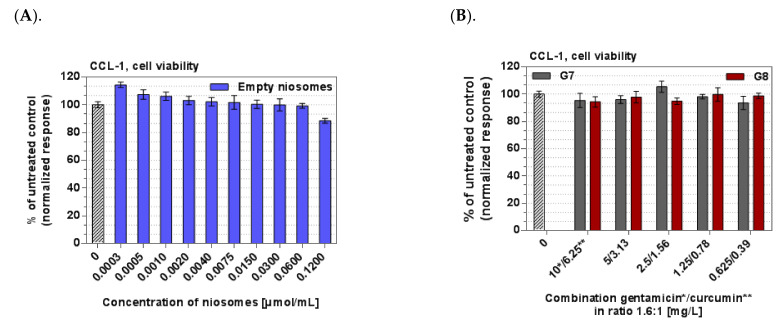
Viability of CCL-1 cells. Legend: (**A**). After exposure to solution containing empty niosomes (30 µmol/mL total lipid). (**B**). After exposure to hybrid in situ niosomal gels based on simultaneously loaded Curc/GS niosomes.

The cytotoxicity potential of the optimal empty or Curc/GS-co-loaded niosomal suspensions and the hybrid thermosensitive in situ gels thereof were tested on CCL-1 normal mice fibroblasts. The results from the in vitro cytotoxicity assay on non-malignant CCL-1 cells revealed that the empty niosomes and the hybrid niosomal in situ gels are devoid of cytotoxicity in the tested concentration range of 0.003–0.12 µmol/mL for total lipid, 0.39–6.25 mg/L curcumin, and 0.625–10 mg/L gentamicin (Figure 10A,B) The maximal tolerated concentrations are above the highest tested concentrations of the applied concentration range. No significant differences to the untreated control were measured.

**Figure 11:**
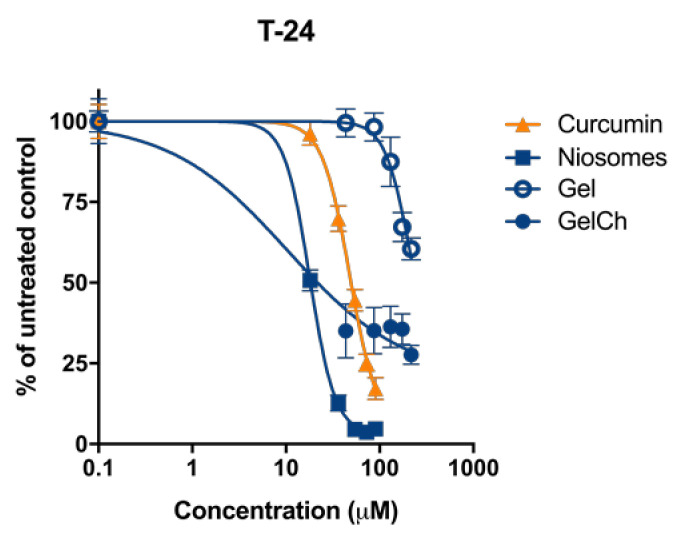
Concentration–response curves of Curcumin and its formulations against T-24 cells evaluated by MTT assay followed 72 h continuous exposure. SD ± represent mean value of 8 independent experiments.

The growth-inhibitory effect of Curc/GS sulfate-co-loaded niosomes and their in situ gels counterparts were tested in comparative way vs. free curcumin against malignant T-24 human tumor cells. The concentration–response curves are shown on Figure 11, and the derived IC_50_ concentrations are presented in Table 5. Evident from the results, the in vitro *c*ytotoxicity of the free gentamicin sulfate was undetectable (>1000 μM) in the tested concentration range and excluded as a factor in the nanocarrier’s cancer growth-inhibitory activity. Therefore, the antitumor activity of the tested systems was evaluated and interpreted in terms of their curcumin content.

As evidenced by the experimental data, curcumin performance was affected by the formulation properties. In the epithelial cells (T-24), a significant improvement in antitumor efficacy for niosomal curcumin was observed, resulting in 2.7-fold decrease in IC_50_ values as compared to the free drug (Table 5). The formulation of curcumin-loaded niosomes into hybrid in situ gels without chitosan, results in an increase in IC_50_ values more than five times as compared to niosomes in suspension. Contrarily, the antiproliferative efficacy of the curcumin formulated in hybrid niosomal in situ gel containing 10% chitosan was 1.7- and 4.8-fold higher as compared to niosomal and free curcumin respectively. Generally, the observed cytotoxicity trends of the nanocarriers are well in accordance with the release kinetics of their cargo described in Section 3.3. As indicated, the drug’s dissolution and diffusion in the gel structure are significantly slowed down, leading to diminished concentration of the free curcumin in cell media and consequently at the site of action. On the other hand, the bioactivity of the niosomal formulation and in situ gel containing chitosan as compared to the referent carrier-free curcumin was even superior in the T-24 cell line, demonstrating the beneficial effect of the tested nanoformulations.

The favorable results obtained in the bladder carcinoma cell line T-24 is of particular importance in view of the potential intravesical therapeutic application of the curcumin- and gentamicin-loaded nanocarriers. Furthermore, the most pronounced growth-inhibitory effect achieved the chitosan gel formulation that, by design, due to the presence of chitosan, is presumed to possess stronger adhesive properties and the ability to reversibly open the tight junction that facilitates the permeation of drugs [57], and possibly is of relevance to the suppressed proliferation of the adherent tumor T-24 cells

## 4. Conclusions

Hybrid in situ gels based on curcumin–gentamicin sulfate-co-loaded niosomes and temperature sensitive Poloxamer 407 and 188 were elaborated and evaluated as a potential drug delivery system for intravesical instillation. Formulations containing 20% *w/w* P407 and 8% *w/w* P188 with or without the addition of 10% chitosan solution were selected as most suitable for intravesical instillation as they showed an optimal phase transition temperature of 31 °C and 27 °C, short gelation time up to 35 s, and a typical rheological response of hard gels at body temperature. The established synergistic antibacterial activity of the active ingredients together with the favorable sustained release patterns and gel erosion kinetics give us the reason to consider the presented hybrid systems as a suitable platform for intravesical co-delivery of curcumin and gentamicin.

## Figures and Tables

**Table 1:** Composition, physicochemical characteristics, and encapsulation efficacy of curcumin and gentamicin sulphate simultaneously loaded niosomes prepared by the thin film hydration method (TFH). In each of the formulations the concentration of gentamicin sulphate hydration solution is kept constant (26.86 µmol/mL), while the CURC content varies from 1.5 µmol/mL (at Curc:SF 1:20) to 3 µmol/mL (at Curc:SF 1:10).

Sample	SF:Chol (mol:mol)	CURC:SF (mol:mol)	HLB	d_h_ (nm) ± SD	PDI ± SD	ζ-Potential (mV) ± SD	EE (%) ± SD	
							Curc	GS
S1	Sp80:Chol 7:3	1:10	4.3	264.3 ± 5.2	0.37 ± 0.04	−10.7 ± 0.7	27 ± 1.6	10 ± 0.8
S2	Sp60:Chol 7:3	1:10	4.7	287.8 ± 3.9	0.41 ± 0.02	−11.2 ± 1.4	24 ± 1.9	12 ± 1.1
S3	Tw60:Chol 7:3	1:10	14.9	25.6 ± 2.2	0.25 ± 0.05	−10.2 ± 1.1	-	-
S4	Sp60:Tw60: Chol3.5:3.5:3	1:10	9.8	373.3 ± 9.4	0.44 ± 0.02	−10.9 ± 2.5	65 ± 0.9	36 ± 1.7
S5	Tw60:Chol 1:1	1:10	14.9	362.1 ± 5.3	0.42 ± 0.03	−12.2 ± 2.1	36 ± 2.1	39 ± 1.3
S6	Sp60: Tw60: Chol 3:3:4	1:10	9.8	595.5 ± 8.5	0.39 ± 0.05	−10.2 ± 1.8	42 ± 0.3	34 ± 0.9
S7	Sp60: Tw60: Chol 3:3:4	1:15	9.8	496.5 ± 5.9	0.41 ± 0.06	−11.7 ± 0.6	51 ± 2.1	34 ± 1.5
S8	Sp 60:Tw 60:Chol 3.5:3.5:3	1:20	9.8	401.4 ± 4.3	0.36 ± 0.03	−11.1 ± 1.4	80 ± 1.9	36 ± 1.2
S9	Sp60: Tw60: Chol * 3.5:3.5:3	1:20	9.8	251.2 ± 5.8	0.32 ± 0.01	−13.8 ± 1.3	81 ± 1.4	-
S10	Sp60: Tw60: Chol ** 3.5:3.5:3	-	9.8	489.1 ± 4.8	0.34 ± 0.06	−12.3 ± 2.3	-	-

* Solely curcumin-loaded niosomes; ** non-loaded niosomes.

**Table 2:** Composition and gelation properties of plain and hybrid niosomal in situ gels.

Formulation Code	Water%	Niosomes *% (*w/w*)	P407% (*w/w*)	P188% (*w/w*)	Ch% (*w/w*)	T_sol–gel_ (°C)	Gelation Time (s)	Gel Erosion Time (h)
G1	75	-	25	-	-	<22 #	-	-
G2	80	-	20	-	-	<27 #	-	-
G3	72	-	20	8	-	33 ± 0.7 # 31 ± 0.2 **	10 ± 1.7 #	4 ± 0.2
G4	70	-	20	10	-	40 ± 1.1 #	30 ± 2.1 #	-
G5	62	-	20	8	10	31 ± 0.8 # 27 ± 0.4 **	15 ± 1.0 #	4.5 ± 0.1
G6	52	-	20	8	20	30 ± 1.5 #	30 ± 2.3 #	-
G7	10	62	20	8	-	33 ± 1.1 # 31 ± 0.8**	10 ± 1.4 #35 ± 1.1 **	4 ± 0.2
G8	-	62	20	8	10	30 ± 1.3 # 27 ± 0.3 **	15 ± 1.1 #33 ± 1.0 **	4.5 ± 0.1

* Formulation S8; # Visually observed; ** Values obtained by dynamic rheological measurements.

**Table 3:** Evaluation of physical stability of selected niosomal formulations and in situ thermosensitive gel thereof after storage at 4 °C for one month.

Formulation Code		Size (nm) ± SD	PDI ± SD	T_sol–gel_ (°C) ± SD	EE (%) ± SD	
					Curc	GS
Niosomes in suspension (Formulation S8)	Freshly prepared	401.4 ± 4.3	0.36 ± 0.03	-	80 ± 1.1	36 ± 1.2
	After storage	431 ± 5.2	0.45 ± 0.05	-	77.9 ± 1.5	30.5 ± 1.4
Niosomes in gel (Formulation G7)	Freshly prepared	465 ± 2.3	0.32 ± 0.03	33 ± 1.1	79.5 ± 1.4	36 ± 2.2
	After storage	468 ± 2.2	0.38 ± 0.02	34 ± 0.9	79 ± 1.9	35.8 ± 2.1

**Table 4:** Antibacterial activity of free and formulated into hybrid niosomal in situ gels curcumin and gentamicin sulfate.

Sample	Parameters	*Staphylococcus aureus*, ATCC 29213	*Escherichia coli*, ATCC 35218
Gentamicin, aqueous solution	MIC	0.25 * mg/L	2 * mg/L
Curcumin, ethanol solution	MIC	50 mg/L	n. a.
GS-NGel	MIC	1 mg/L	4 mg/L
Curc-N Gel	MIC	80 mg/L	80 mg/L
Combination effect GS-NGel + CurcNGgel	MIC_C-GG_	0.5 mg/L	2 mg/L
	MIC_C-GC_	0.3 ÷ 5 mg/L	0.3 ÷ 10 mg/L
	FIC_GG_	0.5	0.5
	FIC_GC_	0.0038	0.0038
	∑FIC_GG/GC_	0.5038–synergistic	0.5038–synergistic
	DEHA_C_	1.70%	0.55%
GS-NGel-Ch	MIC	1 mg/L	4 mg/L
Curc-NGel-Ch	MIC	40 mg/L	80 mg/L
Combination effect GS-NGel-Ch + Curc-NGel-Ch	MIC_C-GChG_	0.5 mg/L	2 mg/L
	MIC_C-GChC_	0.3 ÷ 10 mg/L	0.3 ÷ 20 mg/L
	FIC_GChG_	0.5	0.5
	FIC_GChC_	0.0075	0.0038
	∑FIC_GChG/GChC_	0.5075–synergistic	0.5038–synergistic
	DEHA_C_	10.1%	0.82%
Curc/GS-NGel (GS:Curc = 80:50 mg/L = Curc:GS 1:1.6 wt:wt)	MIC	0.5/0.3 mg/L	2/1.25 mg/L
	MBC	4/2.5 mg/L	4/2.5 mg/L
Curc/GS-NGel–Ch (GS/Curc, 80:50 mg/L)	MIC	0.5/0.3 mg/L	1/0.625 mg/L
	MBC	4/2.5 mg/L	2/1.25 mg/L

Legend: MIC—minimal inhibitory concentration; n. a.—not active; * The clinical breakpoint for gentamicin (GS) is 2 mg/L for both bacterial species; MIC_C-GG_—MIC of the gelling system with GS in combination; MIC_C-GC_—MIC of the gelling system with curcumin (CURC) in combination; FIC_GG_—fractional inhibitory concentration of the gelling system with GS; FIC_GC_—FIC of the gelling system with Curc; ∑FIC_GG/GC_—the sum of the FIC of GS and Curc in gelling systems; DEHA_C_—dehydrogenase activity of the combination; MIC_C-GChG_—MIC of the chitosan–gelling system with GS in combination; MIC_C-GChC_—MIC of the chitosan–gelling system with Curc in combination; FIC_GChG_—fractional inhibitory concentration of the chitosan–gelling system with GS; FIC_GChC_—FIC of the chitosan–gelling system with Curc; ∑FIC_GChG/GChC_—the sum of the FIC of GS and Curc in chitosan–gelling systems; MBC—minimal bactericidal concentration.

**Table 5:** In vitro cytotoxicity (IC_50_ (µM ± SD)) of the free curcumin and its niosomal and thermosensitive gel formulations in the screened malignant cell line.

Cell Line Compound	T-24
Free curcumin	48.1 ± 5.1
Curc/GS Niosomes	17.9 ± 0.6
Curc/GS NGel	100.1 ± 6.9
Curc/GS NGelCh	10.3 ± 0.5

## Data Availability

Not applicable.
